# Nutrition Education Program and Physical Activity Improve the Adherence to the Mediterranean Diet: Impact on Inflammatory Biomarker Levels in Healthy Adolescents From the DIMENU Longitudinal Study

**DOI:** 10.3389/fnut.2021.685247

**Published:** 2021-07-19

**Authors:** Catia Morelli, Ennio Avolio, Angelo Galluccio, Giovanna Caparello, Emanuele Manes, Simona Ferraro, Antonella Caruso, Daniela De Rose, Ines Barone, Carlo Adornetto, Gianluigi Greco, Stefania Catalano, Sebastiano Andò, Diego Sisci, Cinzia Giordano, Daniela Bonofiglio

**Affiliations:** ^1^Department of Pharmacy, Health, and Nutritional Sciences, University of Calabria, Arcavacata di Rende, Italy; ^2^Centro Sanitario, University of Calabria, Arcavacata di Rende, Italy; ^3^Health Center SRL, Cosenza, Italy; ^4^School of Specialization in Food Sciences, University of Rome Tor Vergata, Rome, Italy; ^5^Department of Clinical and Experimental Medicine, University Magna Graecia, Catanzaro, Italy; ^6^Department of Mathematics, University of Calabria, Arcavacata di Rende, Italy

**Keywords:** Mediterranean diet, physical activity, ferritin, C-reactive protein, body composition parameters, healthy lifestyle

## Abstract

Adherence to Mediterranean diet (MD) and physical activity (PA) in adolescence represent powerful indicators of healthy lifestyles in adulthood. The aim of this longitudinal study was to investigate the impact of nutrition education program (NEP) on the adherence to the MD and on the inflammatory status in healthy adolescents, categorized into three groups according to their level of PA (inactivity, moderate intensity, and vigorous intensity). As a part of the DIMENU (Dieta Mediterranea & Nuoto) study, 85 adolescents (aged 14–17 years) participated in the nutrition education sessions provided by a team of nutritionists and endocrinologists at T0. All participants underwent anthropometric measurements, bio-impedentiometric analysis (BIA), and measurements of inflammatory biomarkers such as ferritin, erythrocyte sedimentation rate (ESR), and C-reactive protein (CRP) levels. Data were collected at baseline (T0) and 6 months after NEP (T1). To assess the adherence to the MD, we used KIDMED score. In our adolescents, we found an average MD adherence, which was increased at T1 compared with T0 (T0: 6.03 ± 2.33 vs. T1: 6.96 ± 2.03, *p* = 0.002), with an enhanced percentage of adolescents with optimal (≥8 score) MD adherence over the study period (T0: 24.71% vs. T1: 43.52%, *p* = 0.001). Interestingly, in linear mixed-effects models, we found that NEP and vigorous-intensity PA levels independently influenced KIDMED score (β = 0.868, *p* < 0.0001 and β = 1.567, *p* = 0.009, respectively). Using ANOVA, NEP had significant effects on serum ferritin levels (*p* < 0.001), while either NEP or PA influenced ESR (*p* = 0.035 and 0.002, respectively). We also observed in linear mixed-effects models that NEP had a negative effect on ferritin and CRP (β = −14.763, *p* < 0.001 and β = −0.714, *p* = 0.02, respectively). Our results suggest the usefulness to promote healthy lifestyle, including either nutrition education interventions, or PA to improve MD adherence and to impact the inflammatory status in adolescence as a strategy for the prevention of chronic non-communicable diseases over the entire lifespan.

## Introduction

Adherence to the Mediterranean diet (MD) and physical activity (PA) in adolescence represent powerful indicators of healthy lifestyles in adulthood (1–3).

The MD, characterized by a high intake of vegetables, fruits, legumes, dairy products, and nuts, a moderate intake of fish and poultry, along with a low intake of red meat, processed foods, and saturated lipids (4), has been accepted as one of the healthiest dietary patterns in the world (5). The inverse association between the MD adherence and a wide range of chronic and metabolic diseases is well-known (6), and it may be, at least in part, attributed to the anti-inflammatory properties of MD components (7). For instance, it has been reported that low adherence to the MD is directly associated with a worse profile of circulating inflammation-related biomarkers (8). Although, the relationship between MD and inflammatory markers within a population of European adolescents has been recently investigated (9), the impact of MD and PA on inflammatory status in healthy adolescents remains to be clarified.

The spectrum of PA is broad, ranging from physical inactivity/sedentary behavior to different levels of PA intensities. Particularly, the sedentary behavior has been reported to be implicated in unhealthy conditions (10), while moderate- to vigorous-intensity PA during adolescence leads to chronic disease benefits that can be attributed to the anti-inflammatory effects of exercise (11–14).

Over the last years, the impact of PA on inflammation has received great attention for the identification of biologically active substances, secreted by myocytes (myokines) after skeletal muscle contraction, which can exert beneficial influence on systemic inflammatory responses, and to have positive health effects (15, 16). It has been widely recognized that among myokines interleukin (IL)-6 is locally produced in working skeletal muscle in larger amounts than any other cytokines, and it can account for the increase in plasma IL-6 during exercise (17). Muscle-derived IL-6, along with other classical pro-inflammatory cytokines, such as tumor necrosis factor (TNF)-α and IL-1β, is accompanied by the secretion of other cytokines, such as IL-10, which can create an anti-inflammatory environment (18–20).

Convincing evidence indicates the need to promote healthy nutrition and PA to achieve benefits in terms of the prevention of risk factors for metabolic and chronic diseases. In this context, a carefully designed nutrition education program (NEP) aiming to enhance the knowledge of the MD principles for empowering adolescents toward healthy dietary habits may represent a useful tool to ensure a better quality of life in adulthood.

Thus, the aim of this longitudinal study was to assess over a 6-month period the impact of NEP and PA on MD adherence and on inflammatory status in a cohort of healthy adolescents of a Mediterranean area.

## Materials and Methods

### Study Population

The DIMENU (Dieta Mediterranea & Nuoto) project was funded by the EU Regional Operational Programme Calabria, Italy (prot. #52243/2017), for investigating the impact of the adherence of MD and PA on health status in a sample of adolescents from Southern Italy. Based on an in-depth collaboration with the public high school “Istituto Istruzione Superiore”—Castrolibero, and three swim and sport centers (sports club in Cosenza, Paola, and Crotone of Calabria Region, Italy), we were able to recruit and to select sedentary adolescents and subjects performing recreational sport activities or competitive sports, between December 2018 and January 2019 (21). The exclusion criteria were cognitive or physical/motor limitation, health-related problems, use of medications, restrictive diet (i.e., hypocaloric, low carbohydrate, and low fat). All participants and their parents received a detailed explanation of study purposes. Prior to the enrolment of adolescents in the DIMENU trial, their parents provided written informed consent. All adolescents were subjected to study visits and data collection at baseline (T0) in which the NEP on MD-related issues and sports nutrition was also included. This study was conducted according to the guidelines laid down in the Declaration of Helsinki and approved by the Ethic Committee of the University of Calabria, Italy (#5727/2018).

### Nutritional History Assessment and Nutrition Education Sessions

To collect the nutritional and medical history, participants were orally interviewed at baseline and after 6 months by a team of nutritionists through a nutritional history record, as previously reported (21). Using the KIDMED test (4, 22), we assessed the adherence to the MD in the study population. The score of MD adherence, ranging from 0 to 12, was based on a 16-point paper questionnaire in which a value of +1 was assigned for the consumption of fruits, vegetables, fish, legumes, whole cereals or grain, nuts, oil, dairy products, and yogurt and a negative value −1 for skipping breakfast, consumption of baked goods, sweets, and going to fast food. At baseline, two 30/40-min education sessions, consisting of seminars and interactive lectures structured to cover knowledge of food sources of macro- and micronutrients included in the healthy eating pattern and benefits of MD (basic nutrition concepts, eat at regular intervals, maintain adequate hydration, healthy food choices), were provided for all participants by study nutritionists (EA, AG, GC, EM, and SF) and by three endocrinologists (DB, SA, and SC). In addition, we have created an official website of DIMENU project (https://www.dimenu.it/) (23) and a Facebook page (https://www.facebook.com/dimenu2019) (24) as innovative ways for assuring additional support and information on MD-related issues to all participants.

### Physical Activity Intensity Levels

The intensity of PA levels was estimated following WHO recommendations (25) as physical inactivity [<3 metabolic equivalents (METs)], moderate-intensity (3–6 METs), and vigorous-intensity PA (>6 METs). Specifically, using a questionnaire to assess PA habits that we have described elsewhere (21), the enrolled adolescents were classified into three groups: physical inactivity (PAi = 23), moderate-intensity PA (Pam = 34) [subjects performing at least 60min daily of bicycling (*n* = 2), dancing (*n* = 2), brisk walking (*n* = 2), gymnastics (*n* = 4), aquatic aerobics (*n* = 3), recreational swimming (*n* = 21)] and vigorousintensity PA (PAv = 28) [subjects engaged in at least 60 min daily of jogging or running (*n* = 2), boxing (*n* = 1), tennis (*n* = 1), soccer (*n* = 2), basketball (*n* = 2), squash (*n* = 1), swimming (*n* = 16), aerobic dancing (*n* = 2), and volleyball (*n* = 1)]. The same PA intensity levels of adolescents were confirmed through the interview at T1.

### Anthropometric Parameters and Bioelectrical Impedance Analysis

A detailed description of the anthropometric measurements and bio-impedentiometric analysis (BIA) performed has been reported elsewhere (21). BIA estimated phase angle (PhA), total body water (TBW), body cell mass (BCM), fat-free mass (FFM), and fat mass (FM). Data obtained by BIA test were analyzed using version 1.2.2.8. of the software Bodygram Plus (Akern Srl; Florence, Italy).

### Biochemical Measurements, Erythrocyte Sedimentation Rate, and Interleukin Assays

In the detailed explanation of study purposes, participants were informed to have not eaten at least for 8 h before blood collection. Additionally, participants were reminded 1 week before the study visit. Venous blood samples were collected at T0 and T1, and in order to obtain serum, samples were centrifuged as previously reported (21). ESR was measured by Wintrobe method. Serum C-reactive protein (CRP) levels were detected by immunonephelometry (GOLDSITE Diagnostics, Inc., Shenzhen, China). Serum ferritin levels were measured by enzyme-linked immunosorbent assay (ELISA) (Monobind Inc., Lake Forest, CA, United States), with a detection sensitivity limit of 0.17 ng/ml. Serum iron was determined on a Konelab 20i Chemistry Analyzer (Thermo Electron Corporation, Vantaa, Finland) according to the standardized procedures (Method Iron “Ferene S,” Sclavo Diagnostics, Siena, Italy). Quantification of interleukins was performed using ELISA kits for human IL-6, human TNF-α, human IL-1β, and human IL-10 (Merck Life Sciences, Darmstadt, Germany). The respective sensitivities of these assays were 1.6 pg/ml for IL-6, 0.2 pg/ml for TNF-α, 0.2 pg/ml for IL-1β, and 2 pg/ml for IL-10.

### Mediterranean Diet Meal Plan

All participants received a personalized MD meal plan according to their different PA intensity levels. During the entire period of the NEP, the nutritionists gave verbal and written dietary instructions on the choice of typical Mediterranean foods. The dietary approach was based on the MD pattern according to the last guidelines (26, 27). Each diet plan provides 15–20% of calories through protein, 45–60% of calories through carbohydrates, and 25–30% of calories through fat, with the respective distribution of macro- and micronutrients according to the different energy expenditure of each subject. We calculated the total daily energy expenditure (TDEE) using the formula: TDEE = Basal Metabolic Rate × Physical Activity Level, as recommended by the Italian Society of Human Nutrition (https://sinu.it/2019/07/09/fabbisogno-energetico-medio-ar-nellintervallo-deta-1-17-anni/) (28). Meals included an abundance of plant food (fruits, vegetables, whole grains, nuts, and legumes); fish, poultry, and eggs in moderate amounts; olive oil as the primary source of fat; low consumption of red meats, saturated fats, and sweets. Meals and food plans were designed using MetaDieta software version 4.2.1. (Meteda S.r.l, Roma, Italy).

### Statistical Analysis

Sample size was calculated by considering the whole adolescent population (aged 14–17 years) living in the Mediterranean area of Calabria region (76,000 adolescents) by fixing the confidence level to 95% and the confidence interval to 10% (https://www.surveysystem.com/sscalc.htm). *Post-hoc* power analysis was performed, by G^*^Power software version 3.1.9.4 (University of Heinrich-Heine, Germany), to evaluate the adequacy of the analyzed sample; the effect size was calculated on a website (https://campbellcollaboration.org/escalc/html/EffectSizeCalculator-SMD1.php) on the mean values and standard deviation obtained at baseline (T0) and after 6-month follow-up (T1) for KIDMED score (*d* = 0.4210), ESR (*d* = 0.3621), and ferritin levels (*d* = 0.6584) using Cohen's *d* formula. Data were analyzed by SigmaPlot for Windows version 12.0 (Systat, San Jose, CA, United States) and reported as the mean and SD. Data normality was verified by Kolmogorov–Smirnov test (with Lilliefors' correction). The statistical differences between variables T0 and T1 were evaluated by using paired *t*-test. Frequencies (%) were used to describe qualitative variables that were graphically represented in radar plots. McNemar's chi-squared test was applied to evaluate the statistical differences. Two-way repeated-measures ANOVA was used to test for significant differences between KIDMED score with respect to NEP and PA and for their interaction. Spearman's correlation test was used to assess the association between variables. Linear mixed-effects models were used to test the association between dependent variables (KIDMED, ferritin, ESR, CRP, and cytokines) and independent variables such as NEP and PA along with a set of anthropometric parameters to improve the robustness of our analyses. In the modeling analysis, the *p*-value was adjusted with the Holm–Šidák method (extension of Holm–Bonferroni method). The latter analyses were carried out in a Python 3 environment taking advantage of the *statsmodels* module. Results are considered statistically significant when *p* < 0.05.

## Results

### Characteristics of Participants

The longitudinal DIMENU project was conducted in 85 adolescents (44 girls and 41 boys) who completed the scheduled monitoring visit at T0 and T1. The enrolment at T0 included 92 adolescents, but seven participants dropped out over the course of the study. Table 1 summarizes the anthropometric characteristics and body composition parameters of 85 subjects categorized according to the intensity level of PA into the following three groups: inactivity (PAi), moderate-intensity (PAm), and vigorous-intensity (PAv) PA at T0 and T1. We observed that either NEP or PA had statistically significant effects on the majority of anthropometric measurements and body composition parameters, while no combined effects were found (Table 1).

**Table 1:** Anthropometric characteristics and body composition parameters of participants according to the three physical activity (PA) groups at baseline (T0) and after 6 months (T1).

		**PAi (*n* = 23 subjects)**	**Pam (*n* = 34 subjects)**	**PAv (*n* = 28 subjects)**	***p*****-value**		
					**NEP**	**PA**	**NEP + PA**
BMI (Kg/m^2^)	T0	24.87 ± 5.53	21.91 ± 2.20	21.80 ± 2.30	**0.007**	**0.001**	0.501
	T1	25.25 ± 5.33	22.31 ± 2.25	21.92 ± 1.88			
PhA (°)	T0	5.85 ± 0.52	6.13 ± 0.83	6.36 ± 0.54	0.051	**0.004**	0.518
	T1	5.87 ± 0.67	6.34 ± 0.72	6.55 ± 0.8			
BCM (%)	T0	52.88 ± 2.71	54.11 ± 4.17	55.39 ± 2.54	0.074	**0.004**	0.466
	T1	52.90 ± 3.59	55.19 ± 3.41	56.19 ± 3.61			
BCM (Kg)	T0	36.34 ± 10.4	32.96 ± 5.43	33.97 ± 5.41	**<0.001**	0.151	0.394
	T1	37.26 ± 10.37	34.51 ± 5.79	35.24 ± 5.18			
FFM (%)	T0	73.62 ± 9.27	78.63 ± 9.01	82.37 ± 7.43	**<0.001**	**<0.001**	0.365
	T1	71.69 ± 7.17	75.98 ± 7.38	81.29 ± 6.38			
FFM (Kg)	T0	49.96 ± 11.3	46.49 ± 7.79	50.35 ± 7.91	0.322	0.159	0.663
	T1	49.91 ± 11.7	46.49 ± 8.40	50.89 ± 7.08			
FM (%)	T0	26.38 ± 9.3	21.37 ± 9.02	17.63 ± 7.43	**<0.001**	**<0.001**	0.380
	T1	28.30 ± 7.17	23.99 ± 7.41	18.70 ± 6.38			
FM (Kg)	T0	18.92 ± 9.84	12.75 ± 5.74	10.87 ± 4.54	**<0.001**	**<0.001**	0.478
	T1	20.51 ± 9.38	14.64 ± 4.74	11.80 ± 4.41			
TBW (%)	T0	54.9 ± 7.54	57.68 ± 6.45	62.62 ± 5.12	**<0.001**	**<0.001**	0.341
	T1	53.2 ± 6.2	54.92 ± 5.67	60.94 ± 5.04			

*Data are presented as mean ± SD. T0, Baseline; T1, 6 months of follow-up; PAi, Physical inactivity; PAm, moderate Physical Activity; PAv, vigorous Physical Activity; NEP, Nutrition Education Program; BMI, body mass index; PhA, phase angle; BCM, body cell mass; FFM, fat-free mass; FM, fat mass; TBW, total body water. The statistical differences were evaluated by two-way repeated-measures ANOVA. In boldface are reported statistically significant values. The effects of Nutrition Education Program (NEP) and PA on them are reported*.

### Impact of NEP and PA on the Adherence to the Mediterranean Diet

Over the study period, we found that adherence to the MD evaluated by KIDMED score increased at T1 compared with T0 (T0: 6.03 ± 2.33 vs. T1: 6.96 ± 2.03, *p* = 0.002) in all adolescents. Based on the KIDMED values, we divided the population into optimal (score ≥8), medium (score 4–7), and poor (score ≤ 3) adherence to the MD (22), and we observed that the proportion of adolescents having optimal adherence to the MD was significantly higher after nutritional intervention with respect to baseline (T0: 24.71% vs. T1: 43.52%, *p* = 0.001).

**Figure 1:**
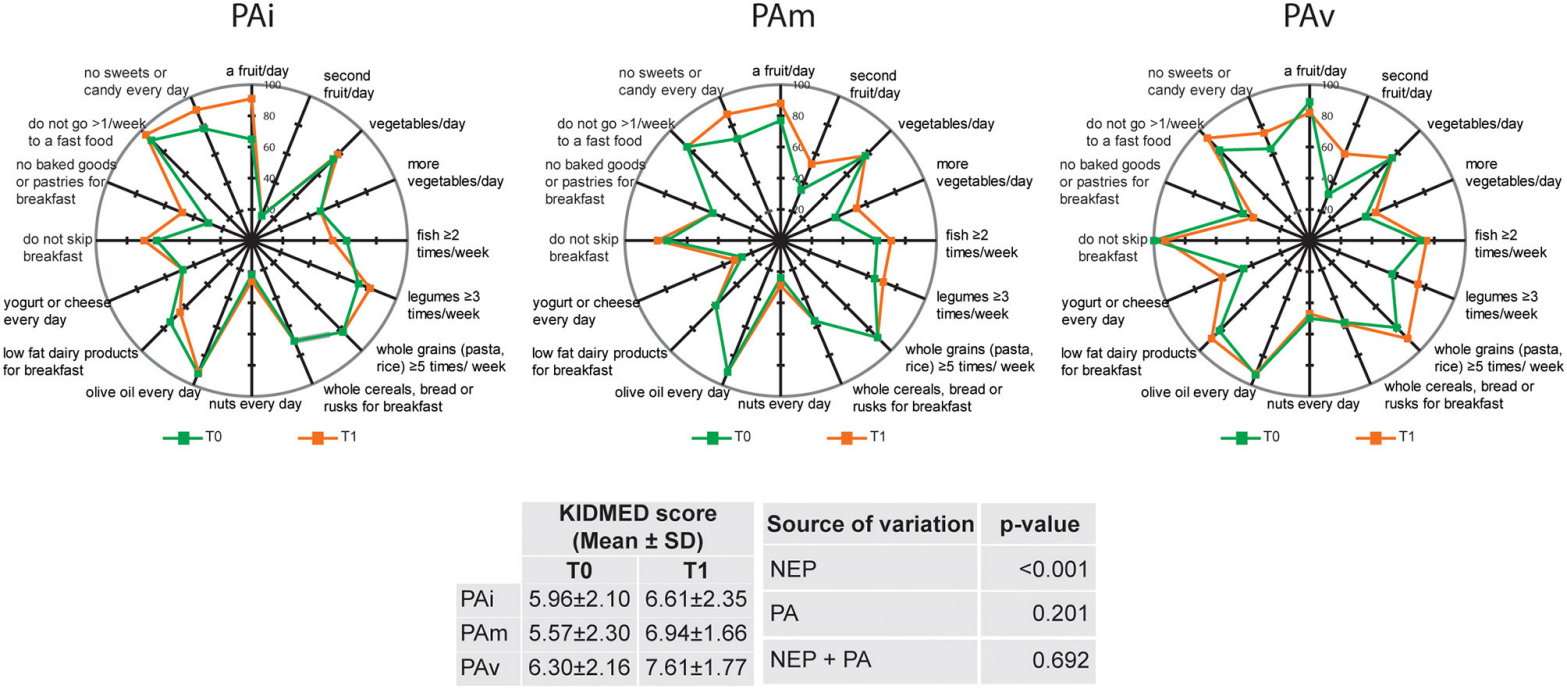
Compliance with items from KIDMED test according to the three physical activity (PA) groups (PAi: inactive; PAm: moderate; PAv: vigorous) at baseline (T0) and after 6 months (T1). The radar chart plots the values of each item of Mediterranean diet score along a separate axis that starts in the center of the chart (0% compliance) and ends at the outer ring (100% compliance). KIDMED score is presented as Mean ± SD; statistical differences were evaluated by two-way repeated-measures ANOVA. NEP, Nutrition Education Program.

In Figure 1, we reported the compliance with items from KIDMED test in the three separate PA groups. No significant changes were observed in the comparison between T0 and T1 for most of items with the exception of an increase in the consumption of “a fruit/day” in PAi group and “second fruit/day” in PAv group (65 vs. 91%, *p* = 0.03 and 32 vs. 60%, *p* = 0.03, respectively). Statistical analyses evidenced that KIDMED score is influenced by NEP (*p* < 0.001) and not by either PA or by NEP+PA (Figure 1).

To evaluate the impact of NEP and PA on the adherence to the MD, we performed linear mixed-effects models including a set of anthropometric parameters (age, gender, weight, height, BMI, and PhA) as independent variables. Results show that NEP had a significant positive effect on the KIDMED score (*p* < 0.001), considering constant all the other variables. Furthermore, PAv had a greater impact on the MD adherence with respect to PAi (*p* = 0.009), while there were no significant differences in KIDMED scores between PAm and PAi (Table 2). To further study these associations, we investigated the interaction between NEP and PA on the KIDMED score, concluding that there were no significant effects between the two variables (Table 2).

**Table 2:** Mixed-effect linear regression model for the association between KIDMED score and NEP, PA, and a set of anthropometric parameters, considering T0 and T1 as a unique longitudinal dataset.

	**Model 1**					**Model 2**				
	**β**	**se**	***p***	**CI (95%)**		**β**	**se**	***p***	**CI (95%)**	
Intercept	−11.924	13.278	0.369	−37.948	14.100	−12.191	13.355	0.361	−38.365	13.984
NEP	0.868	0.218	**0.000**	0.441	1.295	0.463	0.422	0.273	−0.365	1.291
PAm	0.507	0.552	0.359	−0.576	1.590	0.201	0.670	0.764	−1.112	1.514
PAv	1.567	0.599	**0.009**	0.392	2.741	1.099	0.707	0.120	−0.286	2.485
Gender M	−0.443	0.582	0.446	−1.584	0.697	−0.476	0.585	0.416	−1.623	0.671
Age	0.094	0.211	0.655	−0.319	0.508	0.434	0.536	0.418	−0.321	0.506
Weight	−0.076	0.097	0.436	−0.267	0.115	0.677	0.558	0.225	−0.271	0.112
Height	0.099	0.077	0.200	−0.052	0.250	0.093	0.211	0.661	−0.050	0.255
BMI	0.249	0.287	0.384	−0.312	0.811	−0.080	0.098	0.416	−0.302	0.827
PhA	−0.233	0.239	0.330	−0.702	0.236	0.103	0.078	0.186	−0.321	0.506
NEP:PAm						0.262	0.288	0.362	−0.616	1.485
NEP:PAv						−0.247	0.241	0.304	−0.416	1.770

***Model 1:** KIDMED vs. NEP, PA, Gender, Age, Weight, Height, BMI, PhA. **Model 2:** KIDMED vs. NEP, PA, Gender, Age, Weight, Height, BMI, PhA, NEP:PA (Interaction). PAm, moderate physical activity; PAv, vigorous physical activity; CI, confidence interval; The regression coefficient (β), the standard error (se), and the statistical significance (p) are reported. The p-value was adjusted with the Holm–Šidák method (extension of Holm–Bonferroni method). In boldface are reported statistically significant values*.

### Correlations Between Inflammatory Biomarkers and Body Composition Parameters

**Figure 2:**
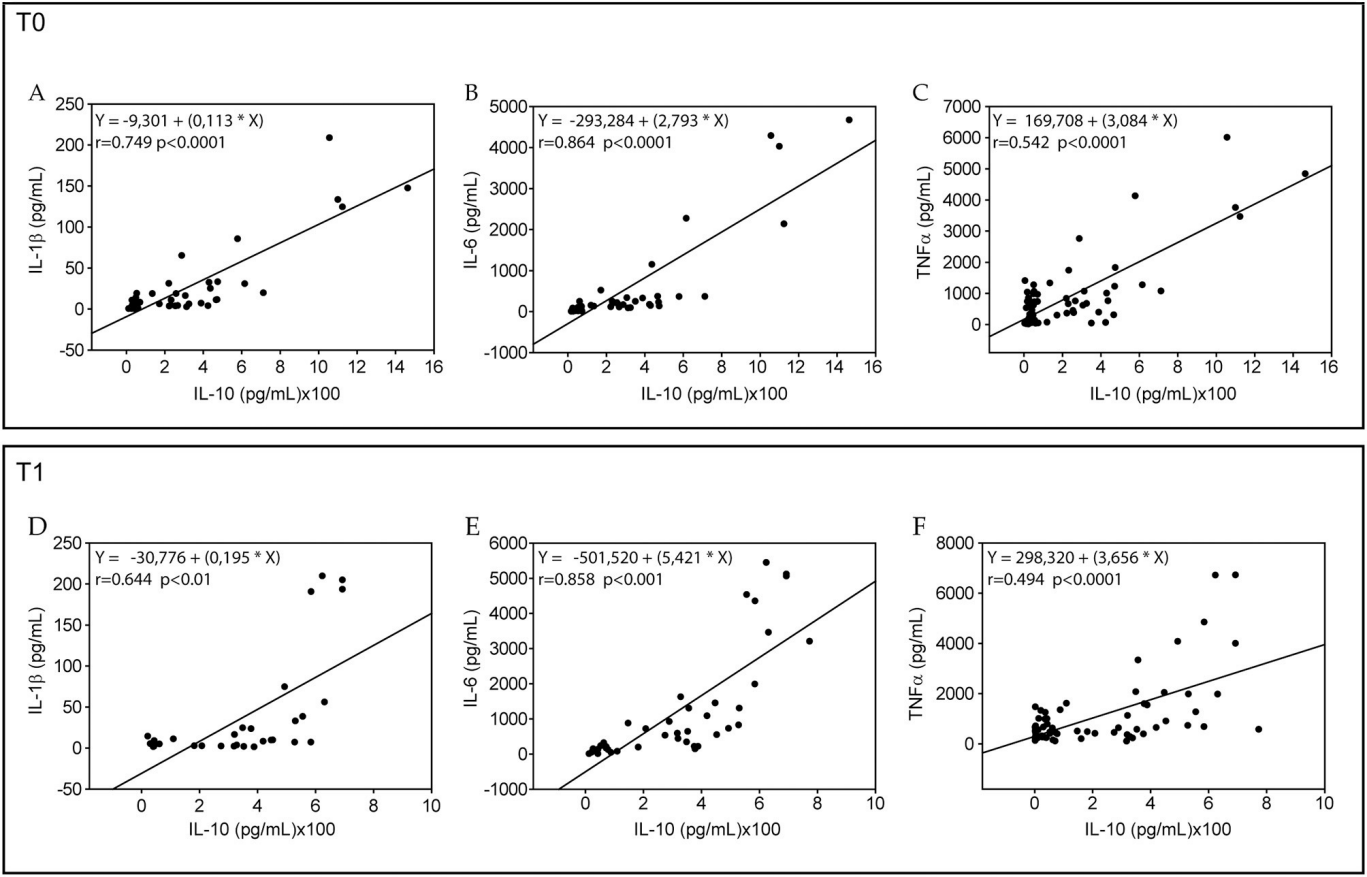
Correlations between interleukin-10 and interleukin-1β, interleukin-6, or tumor necrosis factor (TNF)-α in all sample at baseline (T0) and after 6 months (T1). Association of interleukin (IL)-10 with IL-1β **(A,D)**, IL-6 **(B,E)**, and TNF-α **(C,F)** in all samples at baseline (T0) and after 6 months (T1) were analyzed by Spearman's correlation test. For each linear regression graph, the linear equation (Y), the correlation coefficient (*r*), and the statistical significance (*p*) are reported.

Evaluating the inflammatory status by measuring ferritin, ESR, and CRP along with a panel of serum cytokines in our population longitudinally, we observed that ferritin and CRP levels were significantly reduced in all adolescents (50.66 ± 33.90 vs. 17.79 ± 16.75, *p* < 0.001 and 1.94 ± 3.07 vs. 1.53 ± 2.76, *p* = 0.028, respectively). Using ANOVA, we found that NEP significantly influenced ferritin, ESR, and serum IL-6 as well as TNF-α levels, and PA had effects on ESR, while NEP in combination with PA exerted the effects on serum TNF-α (Table 3). Using the correlation analysis, we found that the pro-inflammatory IL-1β, IL-6, and TNF-α cytokines were significantly associated with the anti-inflammatory IL-10 levels at both times of observation (Figure 2). As expected, serum IL-6, IL-1β, and TNF-α levels were also directly correlated (Supplementary Figure 1).

**Table 3:** Serum inflammatory markers in adolescents according to the three physical activity (PA) groups at baseline (T0) and after 6 months (T1).

		**PAi (*n* = 23 subjects)**	**PAm (*n* = 34 subjects)**	**PAv (*n* = 28 subjects)**	***p*****-value**		
					**NEP**	**PA**	**NEP+PA**
Ferritin (ng/ml)	T0	34.9 ± 19.6	27.7 ± 19.7	31.1 ± 21.3	**<0.001**	0.436	0.701
	T1	21.1 ± 20.5	16.2 ± 15.4	17.0 ± 15.0			
ESR (mm/h)	T0	20.1 ± 10.9	21.1 ± 11.1	12.2 ± 7.1	**0.035**	**0.002**	0.259
	T1	25.9 ± 20.5	21.8 ± 12.7	12.7 ± 8.2			
CRP (mg/L)	T0	2.6 ± 4.5	1.8 ± 2.5	1.62 ± 2.2	0.483	0.133	0.924
	T1	2.4 ± 5.1	1.1 ± 0.5	1.0 ± 0.1			
IL-1β (pg/ml)	T0	9.8 ± 8.9	25.9 ± 40.7	26.9 ± 53.2	0.083	0.152	0.605
	T1	7.9 ± 7.3	26.4 ± 54.6	30.2 ± 63.4			
IL-6 (pg/ml)	T0	124.3 ± 138.4	328.1 ± 930.8	503.7 ± 1170.3	**<0.001**	0.179	0.173
	T1	420.5 ± 456.5	647.0 ± 1321.1	918.1 ± 1592.7			
TNF-α (pg/ml)	T0	552.8 ± 499.4	744.5 ± 993.8	974.7 ± 1567.7	**0.002**	0.239	**0.034**
	T1	665.4 ± 517.8	839.9 ± 917.5	1275.7 ± 1909.6			
IL-10 (pg/ml)	T0	164.65 ± 189.9	180.2 ± 292.1	240.7 ± 360.2	0.449	0.514	0.748
	T1	195.5 ± 184.7	199.3 ± 207.9	268.6 ± 260.5			

*Data are presented as mean ± SD. T0, Baseline; T1, 6 months of follow-up; PAi, Physical inactivity; PAm, Moderate Physical activity; PAv, Vigorous Physical activity; NEP, Nutrition Education Program; ESR, erythrocyte sedimentation rate; CRP, C-reactive protein; IL-1β, interleukin-1beta; IL-6, interleukin-6; TNF-α, tumor necrosis factor alpha; IL-10, interleukin-10. Statistical analysis was performed on ln-normalized samples. The statistical differences were evaluated by two-way repeated-measures ANOVA. In boldface are reported statistically significant values. The effects of Nutrition Education Program (NEP) and PA on them are reported*.

As shown in Table 4, ferritin was positively associated with BCM and FFM (both expressed in kg) at baseline and after 6 months of follow-up. Serum ESR and CRP values were positively correlated with FM (expressed in kg or percentage) and negatively correlated with FFM (expressed in kg or percentage) and TBW at baseline, while serum CRP levels also directly correlated with BMI and inversely with PhA and BCM (expressed in percentage) (Table 4). After 6 months, ESR values showed a positive correlation with FM (expressed in kg or percentage) and an inverse correlation with PhA, TBW, BCM, and FFM (both these latter parameters expressed in kg or percentage), whereas, serum CRP levels were directly correlated with BMI. There were no statistically significant correlations between other inflammatory markers and body composition measurements (data not shown).

**Table 4:** Correlations between serum ferritin, ESR, and CRP levels with body composition parameters in all the sample at baseline (T0) and after 6 months (T1).

		**T0**			**T1**		
		**Ferritin (ng/ml)**	**ESR (mm/h)**	**CRP (mg/L)**	**Ferritin (ng/ml)**	**ESR (mm/h)**	**CRP (mg/L)**
BMI (kg/m^2^)	*r*	0.19	0.189	0.218	0.08	0.073	0.282
	*p*	0.081	0.091	**0.046**	0.460	0.504	**0.009**
Pha (°)	*r*	0.113	−0201	−0.218	0.201	−0.289	−0.036
	*p*	0.304	0.071	**0.046**	0.065	**0.007**	0.740
BCM (%)	*r*	0.110	−0.210	−0217	0.196	−0.273	−0.026
	*p*	0.316	0.060	**0.047**	0.072	**0.011**	0.810
FFM (%)	*r*	−0.002	−0.370	−0.341	0.166	−0.524	−0.066
	*p*	0.983	**0.0007**	**0.0015**	0.129	**<0.00001**	0.547
FM (%)	*r*	0.002	0.370	0.341	−0.167	0.524	0.066
	*p*	0.983	**0.0007**	**0.0015**	0.127	**<0.00001**	0.545
BCM (kg)	*r*	0.311	0.012	0.213	0.315	−0229	0.127
	*p*	**0.004**	0.915	0.052	**0.0035**	**0.035**	0.246
FFM (kg)	*r*	0.277	−0.195	0.04	0.358	−0.484	0.151
	*p*	**0.01**	0.0812	0.717	**0.0008**	**<0.00001**	0.166.
FM (kg)	*r*	0.076	0.384	0.369	−0.047	0.465	−0.0095
	*p*	0.484	**0.0004**	**0.0006**	0.668	**<0.00001**	0.931
TBW (%)	*r*	0.033	−0.392	−0.329	0.119	−0.511	−0.073
	*p*	0.765	**0.0003**	**0.0022**	0.276	**<0.00001**	0.505

*CRP, C-reactive protein; ESR, erythrocyte sedimentation rate; BMI, body mass index; PhA, phase angle; BCM, body cell mass; FFM, fat-free mass; FM, fat mass; TBW, total body water. Data were analyzed by Spearman's correlation test. The correlation coefficient (r) and the statistical significance (p) are reported. In boldface are reported statistically significant values*.

### Impact of NEP and PA on Inflammatory Biomarker Levels

Finally, we investigated the influence of NEP and PA on the levels of inflammatory markers ferritin, ESR, and CRP using linear mixed-effects models with a set of anthropometric parameters (age, gender, weight, height, BMI, and PhA). Table 5 shows that NEP had a negative effect on ferritin (*p* < 0.0001) and CRP (*p* = 0.02) levels. There were no significant effects of PA on the ferritin, ESR, and CRP levels over the study period. NEP and PA interaction had no effects on ferritin, ESR, and CRP (Supplementary Table 1) as well as on cytokine levels (Supplementary Table 2).

**Table 5:** Mixed-effect linear regression model for the association between ferritin, ESR, CRP, and NEP, PA, and a set of anthropometric parameters, considering T0 and T1 as a unique longitudinal dataset.

	**Model 1**					**Model 2**					**Model 3**				
	**β**	**se**	***p***	**CI (95%)**		**β**	**se**	***p***	**CI (95%)**		**β**	**se**	***p***	**CI (95%)**	
Intercept	−248.402	100.597	0.014	−445.5	−51.23	48.384	76.752	0.528	−102.04	198.81	15.745	10.597	0.137	−5.02	36.51
NEP	−14.763	1.516	**0.000**	−17.73	−11.79	2.161	1.421	0.128	−0.62	4.94	−0.714	0.308	**0.020**	−1.31	−0.11
PAm	−4.019	4.658	0.388	−13.14	5.11	0.447	2.783	0.873	−5.00	5.90	0.222	0.426	0.601	−0.61	1.05
PAv	−5.708	5.000	0.254	−15.50	4.09	−4.286	2.983	0.151	−10.13	1.56	0.115	0.453	0.800	−0.77	1.00
Gender M	1.176	4.854	0.809	−8.33	10.69	−9.791	2.864	**0.001**	−15.40	−4.17	−0.184	0.474	0.698	−1.11	0.74
Age	−1.189	1.786	0.506	−4.68	2.31	−0.386	1.071	0.719	−2.48	1.71	0.003	0.161	0.986	−0.31	0.31
Weight	−1.356	0.718	0.059	−2.76	0.05	0.224	0.593	0.706	−0.93	1.38	0.148	0.079	0.060	−0.00	0.30
Height	1.441	0.585	**0.014**	0.29	2.58	−0.116	0.456	0.799	−1.01	0.77	−0.094	0.062	0.127	−0.21	0.02
BMI	5.216	2.120	**0.014**	1.06	9.37	−0.123	1.707	0.943	−3.46	3.22	−0.287	0.233	0.217	−0.74	0.16
PhA	4.680	1.692	**0.006**	1.36	7.99	−1.663	1.354	0.219	−4.31	0.99	−0.163	0.218	0.454	−0.58	0.26

***Model 1:** Ferritin vs. NEP, PA, Gender, Age, Weight, Height, BMI, PhA. **Model 2:** ESR vs. NEP, PA, Gender, Age, Weight, Height, BMI, PhA. **Model 3:** CRP vs. NEP, PA, Gender, Age, Weight, Height, BMI, PhA. PAm, moderate physical activity; PAv, vigorous physical activity. CI, confidence interval; The regression coefficient (β), the standard error (se), and the statistical significance (p) are reported. The p-value was adjusted with the Holm–Šidák method (extension of Holm–Bonferroni method). In boldface are reported statistically significant values*.

## Discussion

In the present study, we highlighted the impact of nutrition education intervention and PA on the adherence to the MD and on inflammatory status in healthy adolescents living in a Mediterranean area of Southern Italy.

In adolescents, Sahingoz and Sanlier (29) have reported that poor nutrition knowledge related to food sources of macro- and micronutrients was associated with low adherence to the MD. Conflicting data are reported in previous observations on the adherence to the MD in adolescent athletes following nutrition education since only in few studies the intervention resulted in improvements in food choices, and it was correlated with most successful sports performance (30–32). Recently, a systematical review of the scientific literature available on MD adherence in adolescents showed the direct association between adherence to the MD and PA (and possibly with diet adequacy) and the inverse correlation with sedentary behavior, while the results for other variables such as gender, age, weight, and socioeconomic status were not consistent (33).

In our population sample, the KIDMED score showed a medium adherence to the MD with the proportion of subjects having an optimal score, which was significantly improved over the study period (T0: 24.7% vs. T1: 43.5%), underlining that they have translated nutrition knowledge into healthier eating habits. This finding is supported by the evidence that using ANOVA NEP had a positive effect on the MD adherence, while PA alone or in combination with NEP did not exert any influence. Using linear mixed-effects models, we confirmed that nutrition education intervention had a positive effect on the adherence of the MD, and interestingly, we were also able to evidence that vigorous PA intensity positively influenced healthy dietary pattern. Surprisingly, the main effects of NEP and PA lose their statistical significance when the model includes the interaction term. We hypothesize that the introduction of the interaction effect increases the model complexity, which would require a larger population sample to potentially obtain significant results. Data on the MD adherence are in agreement with those reported among adolescents and adults living within the Mediterranean area (34–37) as well as in children and adolescents outside of the Mediterranean region (38, 39). Importantly, the positive implications of nutrition education sessions by a team of nutritionists and endocrinologists were observed on increased KIDMED score and a better compliance to the recommendation of the Mediterranean food choices. This seems to be very important at this age range, representing the most appropriate regime for disease prevention (40). Evidence suggests that adhering to the MD and performing regular PA, as an integral part of the traditional Mediterranean lifestyle, have been linked to health benefits (25, 41). For instance, their combined promotion may be a useful means to reduce metabolic risk and obesity in adult populations (42). Specifically, systematic reviews and meta-analyses have reported that high adherence to the MD and PA decreased the risk factors related to several metabolic and chronic diseases, such as type 2 diabetes, metabolic syndrome, neurodegenerative diseases, and cancers, and was associated with a reduced mortality (38, 39). Low-grade inflammation has emerged as critical in the pathogenesis of these chronic diseases being characterized by abnormal cytokine production and the activations of inflammatory signaling pathways (43). The protection provided by the MD pattern against the diseases associated with chronic low-grade inflammation can probably be due to its antioxidant and anti-inflammatory properties (7, 44). Inflammatory process is associated with changes in the production of serum cytokine levels that may either increase (pro-inflammatory) or decrease (anti-inflammatory) in response to inflammation. In our study, serum levels of both pro-inflammatory and anti-inflammatory cytokines were higher in adolescents at T1 compared with T0 irrespective of different levels of PA. Moreover, we found a direct correlation between the assayed pro-inflammatory IL-6, IL-1β, and TNF-α with the anti-inflammatory cytokine IL-10, which may be responsible to control the balance of inflammation in healthy population at both times of observation. Thus, we speculate that these findings are not related to the impact of NEP or PA but are probably due to other factors contributing to maintain inflammatory balance in adolescents.

In clinical practice, ESR and CRP biomarkers are widely used for identifying and monitoring individuals with inflammatory systemic activity (45–49). Evidence from previous studies suggests that ESR, when elevated in adolescence, is positively associated with premature mortality, making it a suitable marker of inflammation when collecting large amounts of data (50, 51). In adolescents, CRP is inversely related to vigorous-intensity levels of PA, showing that exercise may protect against low-grade systemic inflammation in youth (48). In a recent cross-sectional design, Cayres et al. (52) demonstrated that in adolescents both previous sports participation and regular engagement in PA are inversely related to CRP levels, indicating that frequency and intensity of sports are important factors associated with CRP levels. In line with these observations, we also found that CRP and ESR levels were correlated with several body composition parameters indicative of healthier status, CRP levels were significantly reduced over the study period, while ESR was influenced by either PA or NEP in our adolescents. In the past couple of decades, also ferritin, first discovered as an iron storage protein, has been considered a marker of inflammation, used for predicting and even diagnosing many chronic diseases, including cardiovascular disease, diabetes, and various forms of cancer (49, 53–55). Particularly, elevated serum ferritin levels have been demonstrated to be associated with worse metabolic profile and with an increased cardiometabolic risk in adolescence (49), indicating ferritin as a marker of inflammatory-related diseases. Our results showed that ferritin levels were significantly decreased in all adolescents longitudinally. This latter finding is in agreement with a recent investigation in which ferritin was positively and longitudinally associated with worse health conditions at the adolescent stage (55). Finally, NEP was inversely correlated with both CRP and ferritin levels, suggesting the usefulness to measure these inflammatory biomarkers in the young population from a clinical health perspective.

The limitations of the study include the lack of a control group, the relatively small population studied, and the evaluation of the adherence of MD using only KIDMED test. On the other hand, our longitudinal study strengthens the importance to improve healthy dietary habits in adolescents generating useful data for further investigations in a large number of individuals.

## Conclusions

In summary, this longitudinal study demonstrates the effectiveness to improve nutrition education and to foster an adequate PA intensity levels in adolescents in order to enhance MD adherence and to impact inflammatory marker levels as a strategy for assuring the prevention and control of chronic non-communicable diseases over the entire lifespan.

## Data Availability Statement

The raw data supporting the conclusions of this article will be made available by the authors, without undue reservation.

## Ethics Statement

The study involving human participants was conducted according to the guidelines of the Declaration of Helsinki, and approved by the Ethic Committee of the University of Calabria, Italy 384 (#5727/2018). Written informed consent was provided by the participants' legal guardian/next of kin.

## Author Contributions

CM and EA involved in data collection, study design, analysis and interpretation of data, and writing the manuscript. AG, GC, EM, and SF performed nutritional analyses. AC and DD performed blood analyses. IB, SC, and SA performed analyses and involved in the interpretation of data. CA and GG performed statistical analyses. DS, CG, and DB involved in conception of idea, study design, and reviewing manuscript. All authors have read and agreed to the published version of the manuscript.

## Conflict of Interest

EA, AG, GC, EM, and SF were employed by the company Health Center srl. The remaining authors declare that the research was conducted in the absence of any commercial or financial relationships that could be construed as a potential conflict of interest.
