# Supplementary material for: Nutrition Education Program and Physical Activity Improve the Adherence to the Mediterranean Diet: Impact on Inflammatory Biomarker Levels in Healthy Adolescents From the DIMENU Longitudinal Study
Source: Front Nutr. 2021 Jul 19;8:685247. doi: 10.3389/fnut.2021.685247 (PMC8326330; doi:10.3389/fnut.2021.685247)
Supplement: Supplementary file 1 [file Table_1.DOCX]

**Table 1S.** Mixed-effect linear regression model for the association between Ferritin, ESR, CRP and NEP, PA and a set of anthropometric parameters, considering T0 and T1 as a unique longitudinal dataset.

|  | **Model 1** | | |  | **Model 2** | | |  | **Model 3** | | |
| --- | --- | --- | --- | --- | --- | --- | --- | --- | --- | --- | --- |
|  | ***β*** | ***se*** | ***p*** |  | ***β*** | ***se*** | ***p*** |  | ***β*** | ***se*** | ***p*** |
| **Intercept** | -240.915 | 101.360 | 0.017 |  | 35.500 | 78.416 | 0.651 |  | 19.784 | 9.430 | 0.036 |
| **NEP** | **-14.985** | **2.948** | **<0.001** |  | **6.085** | **2.889** | **0.035** |  | **-1.340** | **0.662** | **0.043** |
| **PAm** | -5.055 | 5.743 | 0.379 |  | 0.995 | 3.003 | 0.740 |  | -0.387 | 0.788 | 0.623 |
| **PAv** | -5.286 | 6.044 | 0.382 |  | -4.015 | 3.242 | 0.216 |  | -0.423 | 0.816 | 0.604 |
| **Gender M** | 1.576 | 4.892 | 0.747 |  | **-9.501** | **2.938** | **0.001** |  | -0.135 | 0.412 | 0.743 |
| **Age** | -1.169 | 1.783 | 0.512 |  | -0.491 | 1.114 | 0.659 |  | 0.054 | 0.142 | 0.703 |
| **Weight** | -1.320 | 0.722 | 0.068 |  | 0.131 | 0.606 | 0.829 |  | **0.174** | **0.070** | **0.013** |
| **Height** | **1.399** | **0.589** | **0.018** |  | -0.026 | 0.473 | 0.956 |  | **-0.122** | **0.056** | **0.029** |
| **BMI** | **5.090** | **2.135** | **0.017** |  | 0.056 | 1.736 | 0.974 |  | -0.348 | 0.205 | 0.089 |
| **PhA** | **4.655** | **1.706** | **0.006** |  | -1.519 | 1.372 | 0.268 |  | -0.171 | 0.196 | 0.383 |
| **NEP: PAm** | 1.105 | 3.733 | 0.767 |  | -5.670 | 3.643 | 0.120 |  | 0.875 | 0.843 | 0.300 |
| **NEP: PAv** | -0.613 | 3.890 | 0.875 |  | -4.779 | 3.739 | 0.201 |  | 0.806 | 0.866 | 0.352 |

**Model 1:** Ferritin *vs* NEP, PAm, PAv, Gender, Age, Weight, Height, BMI, PhA, NEP:PA (Interaction)

**Model 2:** Erythrocyte Sedimentation Rate *vs* NEP, PAm, PAv, Gender, Age, Weight, Height, BMI, PhA, NEP:PA (Interaction)

**Model 3:** C-Reactive Protein *vs* NEP, PAm, PAv, Gender, Age, Weight, Height, BMI, PhA, NEP:PA (Interaction)

The regression coefficient (β), the Standard Error (*se*) and the statistical significance (p) are reported. NEP: Nutritional Educational Program; PAm: moderate Physical Activity; PAv: vigorous Physical Activity; BMI: Body Mass Index; PhA: Phase Angle

Note: In bold are reported statistically significant values.
