# Supplementary material for: Nutrition Education Program and Physical Activity Improve the Adherence to the Mediterranean Diet: Impact on Inflammatory Biomarker Levels in Healthy Adolescents From the DIMENU Longitudinal Study
Source: Front Nutr. 2021 Jul 19;8:685247. doi: 10.3389/fnut.2021.685247 (PMC8326330; doi:10.3389/fnut.2021.685247)
Supplement: Supplementary file 2 [file Table_2.DOCX]

**Table 2S.** Mixed-effect linear regression model for the association between IL-1β, IL-6, TNFα, IL-10 and NEP, PA and a set of anthropometric parameters, considering T0 and T1 as a unique longitudinal dataset.

|  | **Model 1** | | |  | **Model 2** | | |  | **Model 3** | | |  | **Model 4** | | |
| --- | --- | --- | --- | --- | --- | --- | --- | --- | --- | --- | --- | --- | --- | --- | --- |
|  | ***β*** | ***se*** | ***p*** |  | ***β*** | ***se*** | ***p*** |  | ***β*** | ***se*** | ***p*** |  | ***β*** | ***se*** | ***p*** |
| **Intercept** | -534.347 | 231.501 | 0.021 |  | 1.497.659 | 6910.572 | 0.828 |  | 2.805.167 | 6514.465 | 0.667 |  | 684.615 | 1628.025 | 0.674 |
| **NEP** | 1.105 | 3.897 | 0.777 |  | 192.184 | 163.278 | 0.239 |  | 114.437 | 111.460 | 0.305 |  | -9.832 | 42.671 | 0.818 |
| **PAm** | 1.674 | 12.355 | 0.892 |  | 122.310 | 294.774 | 0.678 |  | 335.399 | 375.323 | 0.372 |  | 31.747 | 96.603 | 0.742 |
| **PAv** | 13.921 | 13.190 | 0.291 |  | 338.410 | 309.903 | 0.275 |  | 495.626 | 393.785 | 0.208 |  | 98.990 | 100.763 | 0.326 |
| **Gender M** | -4.362 | 9.063 | 0.630 |  | -50.963 | 244.322 | 0.835 |  | -15.605 | 265.029 | 0.953 |  | -8.138 | 76.200 | 0.915 |
| **Age** | -3.605 | 4.825 | 0.455 |  | 7.898 | 106.820 | 0.941 |  | -32.182 | 141.436 | 0.820 |  | 1.330 | 28.612 | 0.963 |
| **Weight** | **-4.214** | **1.638** | **0.010** |  | 12.238 | 53.392 | 0.819 |  | 41.037 | 46.062 | 0.373 |  | 4.926 | 11.637 | 0.672 |
| **Height** | **3.618** | **1.399** | **0.010** |  | 1.978 | 42.488 | 0.963 |  | -15.540 | 39.273 | 0.692 |  | -3.411 | 9.722 | 0.726 |
| **BMI** | **11.115** | **4.601** | **0.016** |  | -72.025 | 148.635 | 0.628 |  | -91.124 | 129.433 | 0.481 |  | -6.868 | 34.224 | 0.841 |
| **PhA** | 2.604 | 2.432 | 0.284 |  | -143.728 | 82.380 | 0.081 |  | 41.632 | 69.257 | 0.548 |  | -21.612 | 24.122 | 0.370 |
| **NEP: PAm** | 0.543 | 4.826 | 0.910 |  | 200.245 | 205.672 | 0.330 |  | -21.617 | 137.873 | 0.875 |  | 0.720 | 53.642 | 0.989 |
| **NEP: PAv** | 3.773 | 5.049 | 0.455 |  | 257.180 | 210.762 | 0.222 |  | 148.192 | 141.890 | 0.296 |  | -24.423 | 54.994 | 0.657 |

**Model 1:** IL-1β *vs* NEP, PAm, PAv, Gender, Age, Weight, Height, BMI, PhA, NEP:PA (Interaction)

**Model 2:** IL-6 *vs* NEP, PAm, PAv, Gender, Age, Weight, Height, BMI, PhA, NEP:PA (Interaction)

**Model 3:** TNFα *vs* NEP, PAm, PAv, Gender, Age, Weight, Height, BMI, PhA, NEP:PA (Interaction)

**Model 4:** IL-10 *vs* NEP, PAm, PAv, Gender, Age, Weight, Height, BMI, PhA, NEP:PA (Interaction)

The regression coefficient (β), the Standard Error (*se*) and the statistical significance (p) are reported. NEP: Nutritional Educational Program; PAm: moderate Physical Activity; PAv: vigorous Physical Activity; BMI: Body Mass Index; PhA: Phase Angle

Note: In bold are reported statistically significant values.
