# Supplementary material for: Nutrition Education Program and Physical Activity Improve the Adherence to the Mediterranean Diet: Impact on Inflammatory Biomarker Levels in Healthy Adolescents From the DIMENU Longitudinal Study
Source: Front Nutr. 2021 Jul 19;8:685247. doi: 10.3389/fnut.2021.685247 (PMC8326330; doi:10.3389/fnut.2021.685247)
Supplement: Supplementary file 3 [file Data_Sheet_1.DOCX]

**Supplementary Figure 1. Correlations between Interleukins in all the sample at baseline (T0) and after six months (T1).** Association of Interleukin (IL)-1β with IL-6 (A and D) or Tumor necrosis factor (TNF)-α (B and E) and IL-6 with TNF-α (C and F) in all the sample at baseline (T0) and after six months (T1) were analysed by Spearman’s correlation test. For each linear regression graph, the linear equation (Y), the correlation coefficient (r) and the statistical significance (p) are reported.
