# Supplementary figures and images for: Nutrition Education Program and Physical Activity Improve the Adherence to the Mediterranean Diet: Impact on Inflammatory Biomarker Levels in Healthy Adolescents From the DIMENU Longitudinal Study
Source: Front Nutr. 2021 Jul 19;8:685247. doi: 10.3389/fnut.2021.685247 (PMC8326330; doi:10.3389/fnut.2021.685247)

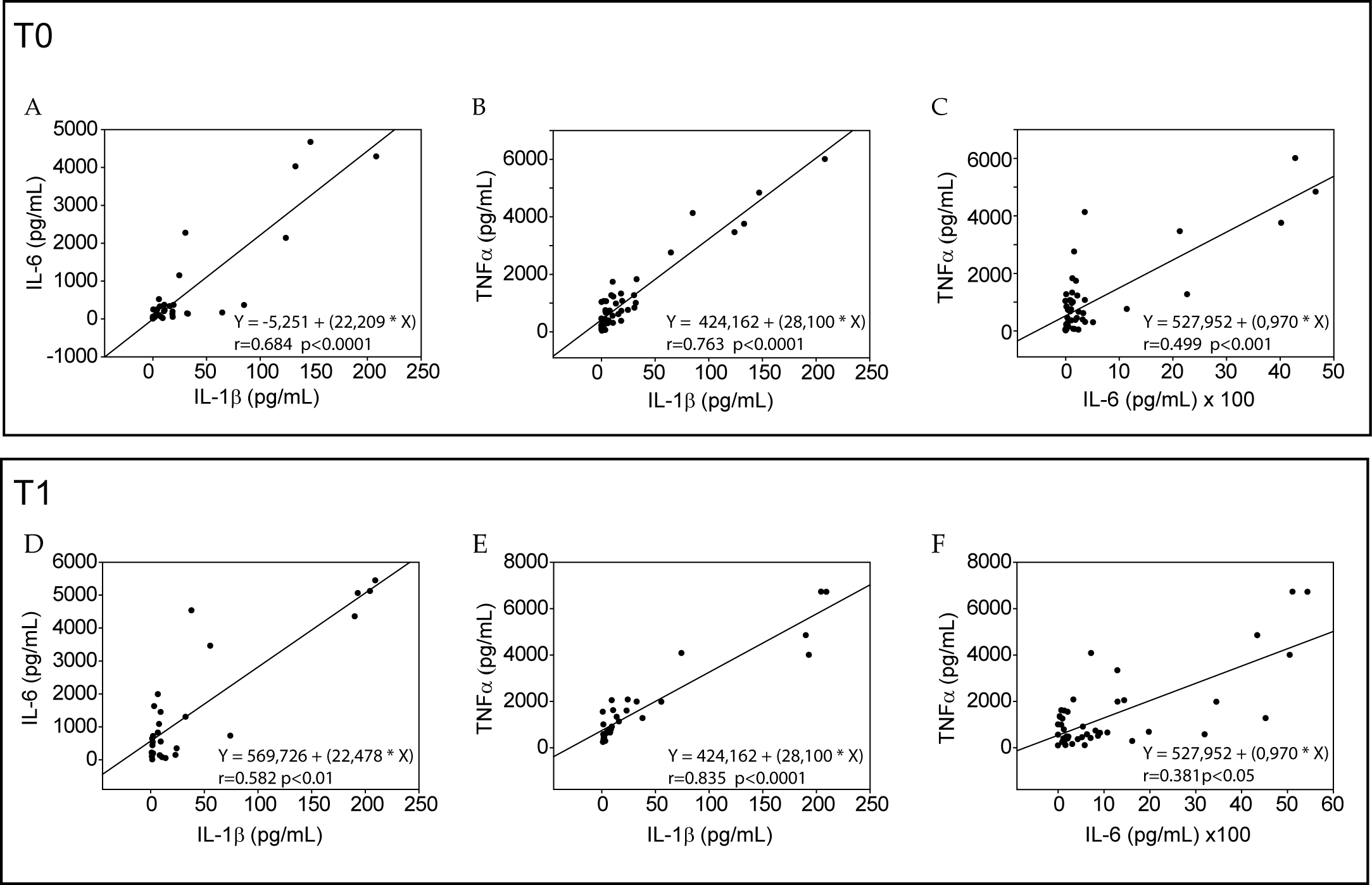

Supplement: Supplementary file 4 [file Figure_1.TIF]
